# An Activity-Aware Sampling Scheme for Mobile Phones in Activity Recognition

**DOI:** 10.3390/s20082189

**Published:** 2020-04-13

**Authors:** Zhimin Chen, Jianxin Chen, Xiangjun Huang

**Affiliations:** College of Telecommunications and Information Engineering, Nanjing University of Posts and Telecommunications, Nanjing 210003, China; 1218012506@njupt.edu.cn (Z.C.); 1219012836@njupt.edu.cn (X.H.)

**Keywords:** activity recognition, machine learning, feature selection, power consumption

## Abstract

In recent years, sensors in smartphones have been widely used in applications, e.g., human activity recognition (HAR). However, the power of smartphone constrains the applications of HAR due to the computations. To combat it, energy efficiency should be considered in the applications of HAR with smartphones. In this paper, we improve energy efficiency for smartphones by adaptively controlling the sampling rate of the sensors during HAR. We collect the sensor samples, depending on the activity changing, based on the magnitude of acceleration. Besides that, we use linear discriminant analysis (LDA) to select the feature and machine learning methods for activity classification. Our method is verified on the UCI (University of California, Irvine) dataset; and it achieves an overall 56.39% of energy saving and the recognition accuracy of 99.58% during the HAR applications with smartphone.

## 1. Introduction

Human activity recognition (HAR) is widely applied in the fields of health care, rehabilitation engineering, and human–computer interaction [1]. The first work on HAR dates back to 1995, where the authors tried to detect posture and motion with worn accelerometers [2]. Recently, with the development of mobile technology, smartphones embedded with plenty of sensors have been proven to be possible for applications of HAR [3]. Generally, the human activity is monitored by sensors or video [4]. Video-based HAR mainly focuses on the frames captured by the camera, while sensor-based HAR relies on the data collected by sensors such as accelerometers, gyroscopes, sound sensors, and magnetometers [5,6]. Because of the development of mobile phones, sensor-based activity recognition has attracted a lot of attention. However, the power supply in smartphones constrains its wide application [7]. As we know, the energy consumption of the smartphones usually comes from two aspects, namely: the sampling rate and signal processing. Nowadays, most energy-saving methods for smartphones are based on decreasing the sampling rate [8,9] or reducing the sensors [10]. Although these solutions might achieve energy-saving, they lose recognition accuracy. In this paper, we try to control the sampling rate adaptively, depending on the correlation of activity during HAR. The contributions of this paper can be summarized as follows:

(1) We define an index for the samples in a steady state variance by considering both the correlation of the samples and the sample interval so as to measure the activity changing.
(2) We study the features of the sensor data and use linear discriminant analysis (LDA) in feature selection to improve the recognition accuracy and reduce computation complexity.
(3) We propose a method to collect sensor data adaptively with the human activity depending on the fluctuation of activity accelerations.

The rest of the paper is organized as follows. Section 2 introduces the related work on the machine learning approaches adopted in HAR. In Section 3, we present an adaptive data acquisition model, which depends on the magnitude of the activity changing. Moreover, we define an energy consumption model and introduce the common machine learning methods that can be used for activity recognition. In Section 4, we compare our model with other solutions in terms of the energy-saving and the recognition accuracy on the public HAR dataset. In Section 5, we conclude this paper.

## 2. Related Work

In this chapter, we give an overview of the approaches in HAR, as well as the methods used to save energy in smartphones. 

### 2.1. Activity Recognition Methods

Machine learning has been applied for activity recognition for a long time. Typical machine learning methods, such as support vector machine (SVM), random forest (RF), and decision tree, require feature extraction, and then classification based on the extracted features. Lara and Labrador [5] summarized the conventional pattern recognition (PR) methods by using machine learning in HAR tasks, and many researchers used Weka [11] to try different approaches. Bao and Intille [12] used the fast Fourier transform (FFT)-based feature computation and a decision tree classifier algorithm to detect physical activities using the data acquired from five small biaxial accelerometers worn on different parts of the body. Wang [13] studied the effectiveness of smartphone accelerators and gyroscopes on HAR. Firstly, they extracted a large number of statistical features from the time and frequency domains; then, they proposed a hybrid method of the filter and wrapper method (FW) to select the best feature. Finally, they used machine learning algorithms such as the *k* nearest neighbors (KNN) and naive Bayes (NB) to classify different activities. In recent years, thanks to the advancement of Graphics Processing Unit (GPU) equipment and high-speed parallel computing technology, the deep learning theory has developed quickly. On the other hand, the deep learning method makes it possible to extract features automatically, instead of using hand-crafted machine learning methods. Wang et al. [14] studied and highlighted advances in deep learning methods for sensor-based activity recognition. Their surveys showed that convolutional neural network (CNN)-based methods dominate studies and are better at inferring long-term repetitive activities, while recurrent neural networks (RNNs) are better at recognizing short-term activities. Kotaro et al. [15] proposed a CNN method for HAR that captures the dynamic characteristics of the time series of sensor data. It was found that the performance of dynamic features with CNN is better than the static features with SVM. Both the dynamic and static features of the raw sensor data are used for HAR with a CNN architecture [16]. The results obtained on the public HAR dataset show that the CNN-based model was significantly better than the baseline method. As mentioned above, although CNN-based methods work well on HAR problems, RNN-based work is scarce. Zhu et al. [17] put forward an end-to-end fully connected deep long short-term memory (LSTM) network for bone-based motion recognition. The proposed model helped to learn feature co-occurrences from the skeletal joints automatically and to achieve state-of-the-art performance on multiple datasets. 

### 2.2. Energy-Efficient Activity Recognition

For the energy efficiency of HAR with smartphones, there are limited works. Yurur et al. [18] proposed a discrete-time inhomogeneous hidden semi-Markov model (DT-IHS-MM)-based generic framework, which should save 40% of energy consumption. Gordon et al. [19] tried to save energy by turning off the irrelevant sensors during the activity recognition phase. Ding et al. [20] improved energy efficiency by reducing the computational complexity during the feature selection in the time and frequency domains. Razzaque et al. [21] achieved energy-efficiency by using compressed sensing. Although several methods have been tried, energy consumption in the experiments was still high. In this paper, we try to save energy by adaptive sampling and adding feature selection into machine learning in order to achieve a high accuracy.

## 3. System Framework and Preprocessing

**Figure 1:**
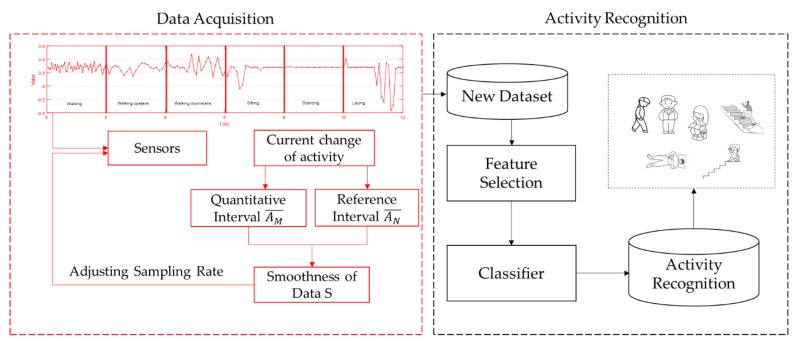
Energy-efficient activity recognition system.

In this section, we introduce the system. Our system is designed to allow mobile phones to implement activity recognition with less energy consumption—it depicted as in Figure 1, which consists of two parts. One is to preprocess the samples based on the outputs from a three-axis accelerometer during the data acquisition phase. The other part is activity recognition with the three-axis accelerometer and three-axis gyroscope based on the time and frequency domain features.

For outputs from a three-axis accelerometer, they fluctuate with the changing of different activities. However, this kind of fluctuation is regular, and the fluctuation is related to the changing of activities. According to this feature, we reduced the collected samples by removing the similar ones. During activity recognition, we combined feature selection with different classifiers. We chose the linear discriminant analysis (LDA) to select the features to improve the recognition accuracy, and to reduce the computation complexity. We also tried different machine learning classifiers combined with grid search and cross-validation so as to implement activity recognition. Lastly, we could achieve a high recognition accuracy among activities such as walking, walking upstairs, walking downstairs, sitting, lying, and standing.

### 3.1. Data Preprocessing

Compared to the traditional energy-saving methods, we focused on the data acquisition phase. Traditional methods usually collect data at a fixed sampling rate. These methods are convenient, but consume too much energy because of the correlation of the samples. In this paper, we propose an adaptive data acquisition method. When the activity changes significantly, the sampling rate is appropriately increased. Conversely, when the activity changes slowly, the sampling rate will be reasonably reduced.

Therefore, the following questions should be taken into consideration:

(1) How to judge the degree of activity change in data collection?
(2) How to control the change of sampling rate?

**Figure 2:**
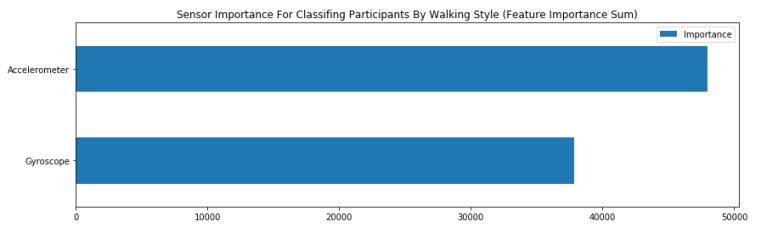
Sensor importance for classifying activities.

In order to solve these problems, we made some definitions. In our system, we used the accelerometer and gyroscope to monitor activities. To find which sensor plays a significant role in activity recognition, we fitted a basic light gradient boosting framework (LGBM) model to the walking data. Light GBM is a gradient boosting framework that uses a tree-based learning algorithm. Figure 2 shows the sensor importance for classifying activities. 

Furthermore, we also compared the sensor importance in other activities in the same way. We found that the accelerometer sensor also plays a more important role. Therefore, accelerometer data were chosen for use in the adaptive sampling phase. Furthermore, in order to measure the fluctuation, we proposed a smoothness index instead of using variance.

The index was used to measure the changing degree of activities, and it provided a basis for the follow-up adjustment of the sampling rate. As we know, the change of variance is less sensitive than the smoothness, and the computation of variance is constrained on a segment of data, without consideration of large intervals. However, the index can represent these features. We calculated the smoothness as follows.

(a) Assume the acceleration from the three-axis acceleration and its average value at time *t* is $A_{i}\left(i=1,2,3\cdots \right)$.
  (1) $$A_{i}=\frac{A_{x}+A_{y}+A_{z}}{3}$$
  In the Equation (1), $A_{x}$, $A_{y}$, and $A_{z}$ represent the acceleration of the x-, y-, and z-axis, respectively. We define $\left|A_{i}-A_{i-1}\right|$ as the current change value of data.
(b) Take period *p*1 as the quantization interval of data change degree. Suppose there are m sampling data in the time interval [t−p1,t], recorded as $A_{j}\left(j=1,2,\cdots ,m\right)$, we define the mean of the quantitative interval $\bar{A_{M}}$ as
  (2) $$\bar{A_{M}}=\frac{\left|A_{m}-A_{m-1}\right|+\left|A_{m-1}-A_{m-2}\right|+\cdot \cdot \cdot +\left|A_{2}-A_{1}\right|}{m}$$
  It is unreasonable to measure the smoothness only by using the change value of the interval $\bar{A_{M}}$. Hence, we also chose a smooth reference interval.
(c) Take another period of time, *p*2, (p2>p1>0) as the reference interval of the data change degree. Suppose there are n sampling data in the time interval [t−p2,t], recorded as $A_{j}\left(j=1,2,\cdots ,n\right)$, we define the mean of the reference interval $\bar{A_{N}}$ as
  (3) $$\bar{A_{N}}=\frac{A_{1}+A_{2}+\cdot \cdot \cdot +A_{n}}{n}$$
(d) After achieving $\bar{A_{M}}$ and $\bar{A_{N}}$, calculate the smoothness index of the data. The definition of the smoothness S of the data is
  (4) $$S=\frac{\bar{A_{M}}}{\bar{A_{N}}}=\frac{n(\left|A_{m}-A_{m-1}\right|+\left|A_{m-1}-A_{m-2}\right|+\cdot \cdot \cdot +\left|A_{2}-A_{1}\right|)}{m\left(A_{1}+A_{2}+\cdot \cdot \cdot +A_{n}\right)}$$

The smoothness index reflects the fluctuation of the data. If the value of smoothness is large, the data fluctuation amplitude is large and the sampling rate should be increased. If the value of smoothness is small, the data fluctuation amplitude is small and the sampling rate should be reduced. After judging the changing degree of data collection, we considered how to control the sampling rate. Finally, we set a reasonable threshold to adjust the sampling rate. Threshold setting is usually determined by the environment. To set an appropriate threshold, we defined four parameters.

Firstly, we defined a basic sampling rate. This sampling rate was determined by the recordings of the dataset. We ensured that the adjusted sampling rate was lower than this value. Secondly, we set a minimum sampling rate for data collection, so as to ensure the adjusted sampling rate was higher than this. Finally, we also set a maximum sampling rate. Moreover, the quantization interval of data *p*_1_ was limited to be larger than the maximum sampling interval. The setting of the sampling rate was determined by the value of smoothness.

### 3.2. Recognition Classifier

In the machine learning algorithm, we used a linear discriminant analysis (LDA) to select the features. LDA is also known as the Fisher discriminant analysis, which reduces the dimension for a more favorable classification. It uses the category markers of the training samples to pursue the projection method that can best separate the data of each category. Supposing there are *C* classes and the *i* example in class *c* is $c_{i}$, *μ* is the average of $c_{i}$. $S_{t}$ is the total scatter matrix, $S_{b}$ is the between-class scatter matrix, $S_{w}$ is the within-class scatter matrix, and $n_{i}$ is the number of categories. *λ* is the undetermined coefficients in the Lagrange multiplier. In order to achieve feature selection, Equations (5)–(7) must be satisfied.

(5) $$S_{w}=\sum_{i=1}^{c}S_{i}$$

(6) $$S_{b}=\sum_{i=1}^{c}n_{i}(c_{i}-\mu ){\left(c_{i}-\mu \right)}^{T}$$

(7) $$S_{b}w_{i}=\lambda S_{w}w_{i}$$

Here, w is the point on the classification lines. From Equations (5)–(7), we can find the features that have the greatest impact on the question.

There are various classifiers on activity recognition, including logistic regression, linear support vector classifier (linear svc), support vector machine (SVM), decision tree (DT), random forest (RF), and gradient boosting (GB). In addition, to make our model more accurate, we used Grid search and cross-validation to achieve automatic adjustment.

### 3.3. Energy Consumption Model

**Figure 3:**
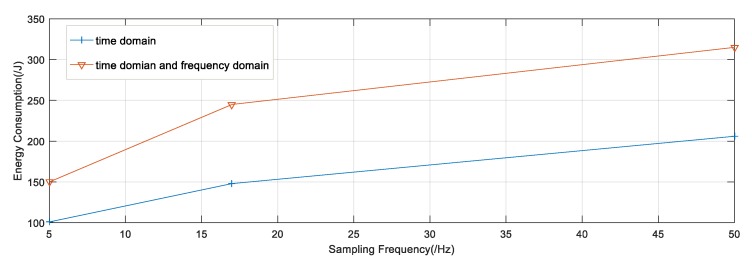
Energy consumption for smartphone between 2 h.

Supposing the consumption of energy is irrelevant to ongoing activities, we studied the relationship between the sampling rate and energy consumption. In addition, the features selected also played an important role in energy consumption. In our dataset, we took the time domain and frequency domain features into consideration. Figure 3 plots the consumption of energy for 2 h on a Samsung Galaxy. During this period, we turned off the network interface and screen display, then used PowerTutor to measure energy consumption.

In general, energy consumption increases with the sampling frequency. However, the increase in energy consumption is non-linear. The computation complexity of the FFT operation results in the non-linear growth. Besides that, there is no specific rule to measure the relationship between the sampling rate and energy consumption. To simplify this problem, we propose that when the sampling frequency is less than 16 Hz, the energy consumption is proportional to the sampling frequency; when the sampling frequency is greater than 16 Hz and less than 50 Hz, the energy consumption is also proportional to the sampling frequency. However, the ratio at this time is less than before. Under this circumstance, we can use the approximate method to calculate energy consumption.

In our model, we assumed that energy consumption was proportional when the sampling rate was less than 16 Hz, and was also directly proportional when the sampling rate was greater than 16 Hz and less than 50 Hz. The proportion is shown in Figure 3.

## 4. Experiment Evaluation

We evaluated our model on the UCI (University of California, Irvine) HAR dataset [22] in terms of accuracy and computational complexity.

### 4.1. UCI HAR Dataset

The dataset consists of recordings about 30 volunteers within an age bracket of 19–48 years old. Each person performed six activities, including walking, walking upstairs, walking downstairs, sitting, standing, and laying, with a smartphone on the waist. The three-axial linear acceleration and three-axial angular velocity data have been recorded at a constant rate of 50 Hz. The sensor signals (accelerometer and gyroscope) were pre-processed by applying noise filters and then sampled in fixed-width sliding windows of 2.56 s with a 50% overlap (128 readings/window). The sensor acceleration signal, which has gravitational and body motion components, was separated using a Butterworth low-pass filter into body acceleration and gravity. The gravitational force is assumed to have only a low rate of components, therefore a filter with a 0.3 Hz cutoff rate was used.

### 4.2. Experiment Environment

The experiment was run on a Mac Pro (ME253CH/A) with an Intel E5 Central Processing Unit (CPU) and the computer’s operating system was Windows 10. The smartphone was a Samsung Galaxy with an android system. Python language was used to implement our algorithm.

### 4.3. Preprocessing Parameters

The experimental parameters were determined based on the previously proposed model in Section 3. Table 1 lists the data preprocessing parameters.

Furthermore, through multiple experiments, we determined the selection threshold for smoothness. The maximum value was determined by the fluctuation of the transition of the activity, and the minimum value was slightly larger than the minimum fluctuation when the activity was stable. What is more, the selection of the intermediate value was dependent on the general distribution of the fluctuation value. Finally, we determined the sampling intervals corresponding to the smoothness intervals. The change of sampling rate is shown in Table 2. There are two main reasons we set the sampling rate. First, we needed to ensure the recognition accuracy while changing the sampling rate. If the sampling rate was too small, the activity recorded was less, and the data put into the model were less too—the data may be over-fitted and the recognition accuracy decreased. While debugging the sampling rate, we used the simple DNN model, because it could better show the recognition accuracy with different rates. 

**Figure 4:**
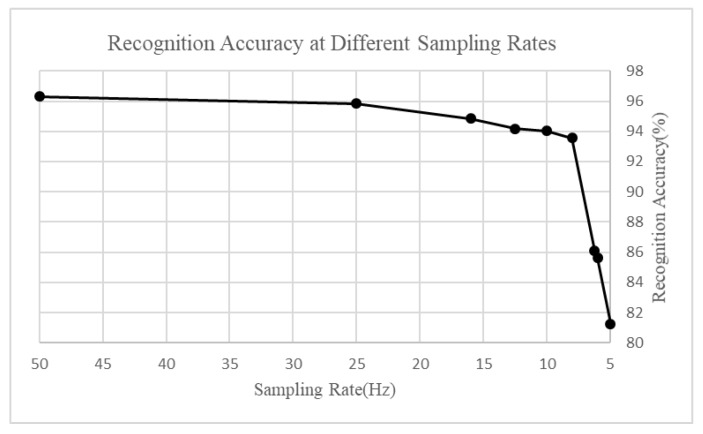
Recognition with a different sampling rate.

Figure 4 depicts the recognition accuracy with different sampling rates. Firstly, we can see that when the sampling rate was less than 8 Hz, the recognition accuracy decreased evidently, so the minimum sampling frequency was selected as 8 Hz, and we still used 50 Hz as the maximum sampling frequency. Secondly, we conducted the experiment repeatedly and found that the data obtained by selecting such a sampling rate were the most suitable. Based on the above reasons, we finally determine the sampling rate of adaptive sampling. 

### 4.4. Training Parameters

In our activity recognition model, we used a variety of classification methods in machine learning for comparison, as well as deep learning models. Table 3 lists the parameters in the activity recognition model. These parameters were determined by grid search and cross-validation. In this way, we could ensure the accuracy of activity recognition.

### 4.5. Results in Activity Recognition

We combined LDA with machine learning, in this way we proved that the feature selection LDA improved our recognition accuracy. Table 4 shows the recognition accuracy in machine learning.

From the table, we can clearly see that feature selection plays an important role in activity recognition. The same can improve the accuracy by about 4%, based on feature selection. In addition, Grid search and cross-validation also improved the accuracy, but increased the computation complexity.

What is more, we compared our method with other state-of-the-art methods in HAR. Table 5 lists the recognition accuracy of different methods in the UCI HAR Dataset, and Table 6 lists the computation complexity in the different methods. From the table, we can see that the accuracy in our method was higher than the traditional machine learning methods with handcrafted features, and was higher than some deep learning methods, such as CNN and RNN methods. The reason our method was better than the others was mainly due to feature selection. A better extraction of the features of the 564-dimensional features has an impact on the results. Furthermore, the use of machine learning has less computation complexity than deep learning methods.

### 4.6. Results in Energy Saving

**Figure 5:**
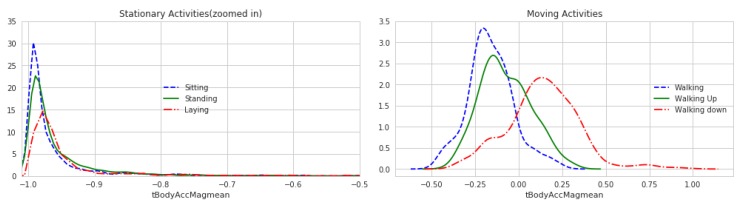
Curve of acceleration change (before preprocessing).

**Figure 6:**
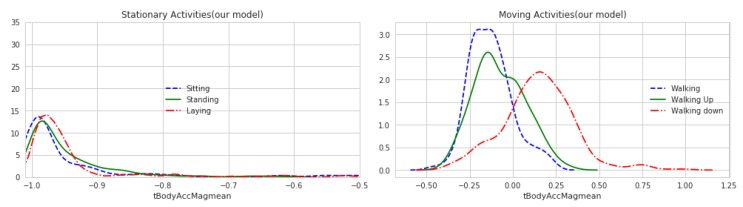
Curve of acceleration change (after preprocessing).

By adjusting the corresponding parameters in our model and running the model, we could compare and prove the rationality of our model through the change degree of the sensor data. From the Figure 5 and Figure 6, we can see the range of acceleration change is roughly similar between before and after preprocessing. Although there are some small changes in the figure, in the recognition process, we synthesized the multi-dimensional features, so as long as the overall trend does not change, the recognition process can still have better results.

**Figure 7:**
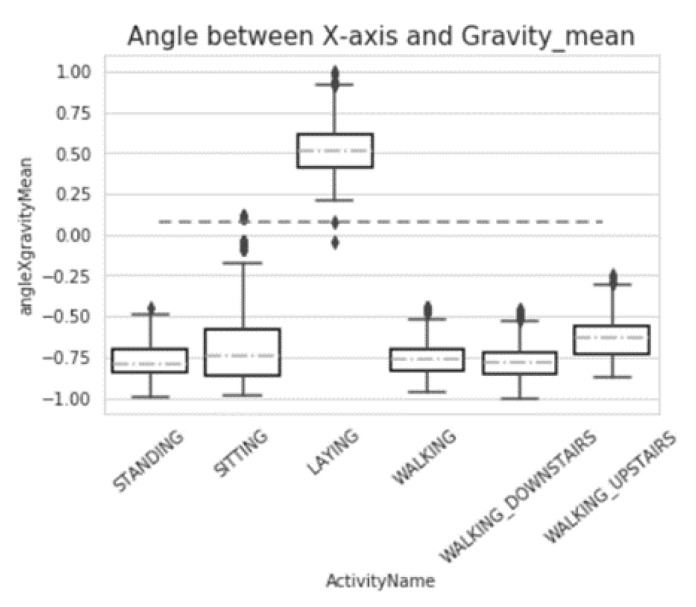
Box-plot of angle between the *x*-axis and Gravity_mean (before preprocessing).

**Figure 8:**
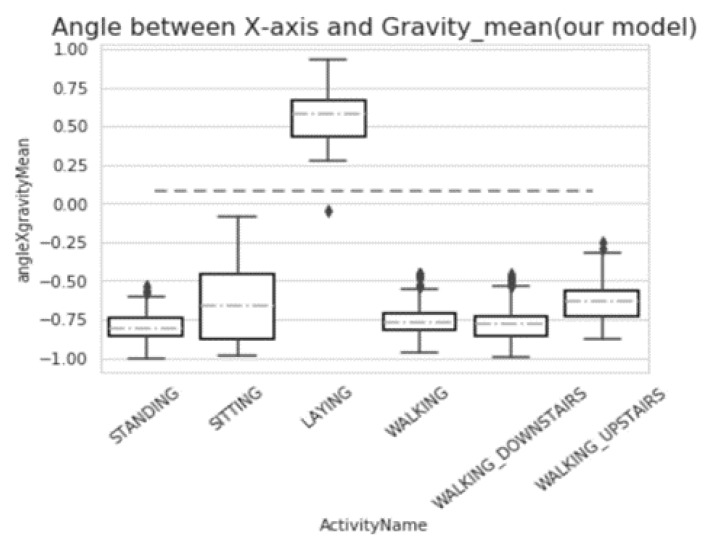
Box-plot of angle between the *x*-axis and Gravity mean (after preprocessing).

From Figure 7 and Figure 8, we can compare the data from the maximum value, minimum value, median, and upper and lower quartile. It is not difficult to see that our model did not affect the overall change of data.

According to the above model for data collection, energy consumption can be greatly saved. Obviously, the dataset size was reduced a lot in our model. Because the acquisition of data requires energy composition, the reduction in data can be seen as a reduction in energy consumption. In the UCI HAR Dataset, 7352 data were recorded, while in our model, only 2569 data were recorded. According to the model proposed in Section 3, we found that in our algorithm, we saved 56.39% energy compared with the fixed sampling method.

Based on energy-saving, we still maintained a high recognition accuracy. In the recognition phase, we not only focused on the accuracy of activity recognition, but also on the computational complexity. Table 7 lists the performance of different machine learning classifiers. It is noted that the accuracy was close to 99.5%. The SVM classifier with a linear kernel had the highest accuracy of 99.58%, and the SVM with an RBF kernel had the second highest activity recognition of 99.49%. Therefore, we can conclude that the SVM classifier with a linear kernel is the best classifier in activity recognition.

**Figure 9:**
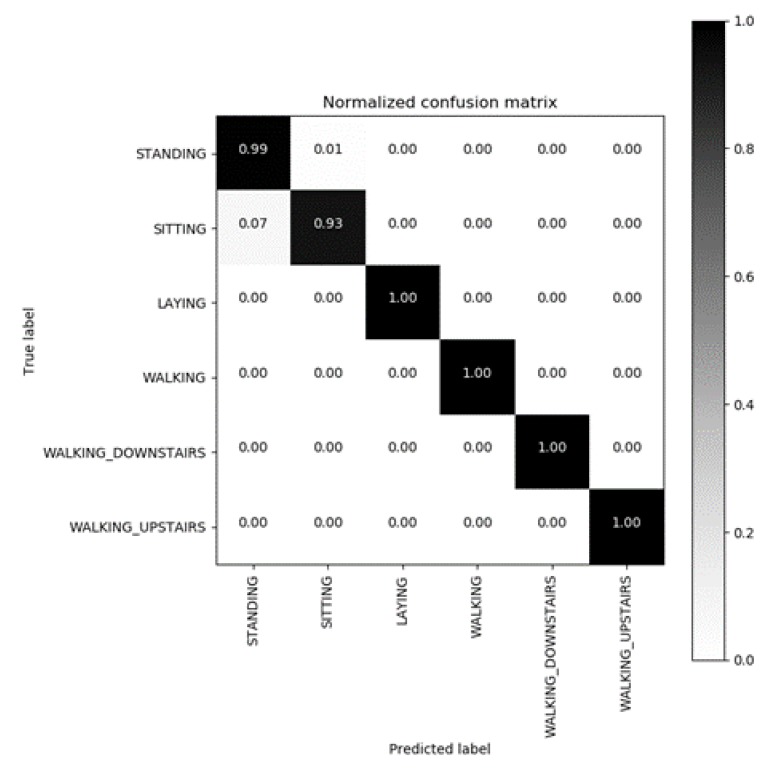
Confusion matrix of a support vector machine (SVM) classifier with linear kernel.

To further explore the accuracy of activity recognition, we drew a confusion matrix to analyze the causes of activity misjudgment. In the confusion matrix (Figure 9), it is easy for us to see the recognition accuracy of each activity.

In this figure, we note that the activity recognition accuracy is close to 1, except for sitting and standing. Sitting and standing can be mistaken for each other; because they are static behaviors, the acceleration tends to be stable, which is difficult to distinguish. Now, we compare our solution with other previous work from the aspect of energy-saving and recognition accuracy.

Table 8 shows the energy-saving and recognition accuracy. From the table, we note that from the aspect of energy-saving and recognition accuracy, our method is better than others.

## 5. Conclusions

With the development of artificial intelligence, people begin to use sensors to monitor activity, and pay more and more attention to energy saving. In this paper, we propose an adaptive data acquisition method to change the sampling rate. This method is based on the fluctuation of the data collected by sensors. We adjust the sampling rate to make sure it is positively correlated with the data fluctuation. What is more, we use LDA to complete feature selection in the recognition phase. Finally, we choose the most suitable classifier according to the computational complexity and accuracy. In this way, we can save energy when achieving activity recognition. Consequently, we can save 56.39% energy and remain activity accuracy at 99.58%.

## Figures and Tables

**Table 1:** Parameters in data acquisition.

Preprocessing Parameters	Value
Basic sampling rate	50 Hz
Maximum sampling rate	50 Hz
Minimum sampling rate	8 Hz
Quantitative interval *p*_1_	0.24 s
Reference interval *p*_2_	0.8 s
	(1)

**Table 2:** Change of sampling rate.

Smoothness Intervals (S)	Sampling Rate
0 < S ≤ 0.04	8 Hz
0.04 < S ≤ 0.08	10 Hz
0.08 < S ≤ 0.12	12.5 Hz
0.12 < S ≤ 0.16	16 Hz
0.16 < S ≤ 0.2	25 Hz
S > 0.2	50 Hz

**Table 3:** Parameters in the machine learning model.

Parameters	Value
Logistic regression	C = 20, penalty = l2
Liner SVC	C = 1, penalty = l2
SVM (rbf kernel)	C = 16, gamma = 0.0078125
Decision tree	Max_depth = 7
Random forest	Max_depth = 11, n_estimators = 170
Gradient boosting	Max_depth = 5, n_estimators = 130

**Table 4:** Recognition accuracy in machine learning.

Classifier	Recognition Accuracy (%)	Recognition Accuracy with LDA (%)	Recognition Times (s)
Logistic Regression	96.3	98.17	6.47
Liner SVC	96.57	98.3	4.73
SVM (rbf kernel)	96.27	98.47	4.82
Decision tree	86.46	97.30	7.21
Random forest	91.31	98.23	7.47
Gradient Boosting	88.86	98.13	12.10

**Table 5:** Classification results of the UCI (University of California, Irvine) dataset.

Methods	Recognition Accuracy (%)
Dynamic time warping [23]	89.00
Handcrafted features + SVM [24]	89.00
Convolutional neural network [25]	90.89
Hidden Markov models [26]	91.76
PCA + SVM [27]	91.82
Stacked autoencoders + SVM [28]	92.16
Hierarchical continuous HMM [28]	93.18
Bidir-LSTM network [29]	93.79
A multi-layer parallel LSTM network [30]	94.34
Convolutional neural network [31]	94.79
Convolutional neural network [16]	95.31
Fully convolutional network [32]	96.32
Genetic algorithm to optimize feature vector [33]	96.38
Bidirectional LSTM network [34]	92.67
CNN [35]	94.00
Hierarchical deep learning model [36]	97.95
Our method (LDA + SVM)	98.47

**Table 6:** Computation complexity.

Method	Recognition Time (s)
Convolutional neural network [31]	21.32
Convolutional neural network [16]	25.61
Multi-layer serial LSTM network	65.02
Multi-layer parallel LSTM network	5.76
Our method (LDA + SVM)	4.82

**Table 7:** Model performance at different classifiers.

Classifier	Recognition Accuracy (%)
Logistic regression	99.38
SVC (linear kernel)	99.58
SVM (RBF kernel)	99.49
Decision tree	99.49
Random forest	98.85

**Table 8:** Comparison with different methods.

Method	Energy-Saving	Recognition Accuracy
Adaptive energy-saving strategy [37]	28%	92%
Adaptive accelerometer-based activity recognition [38]	50%	89%
Our method	56.39%	99.58%
